# Detection of oral squamous cell carcinoma in clinical photographs using a vision transformer

**DOI:** 10.1038/s41598-023-29204-9

**Published:** 2023-02-09

**Authors:** Tabea Flügge, Robert Gaudin, Antonis Sabatakakis, Daniel Tröltzsch, Max Heiland, Niels van Nistelrooij, Shankeeth Vinayahalingam

**Affiliations:** 1 Department of Oral and Maxillofacial Surgery, Charité – Universitätsmedizin Berlin, Corporate Member of Freie Universität Berlin and Humboldt Universität zu Berlin, Augustenburger Platz 1, 13353 Berlin, Germany; 2 Department of Oral and Maxillofacial Surgery, Radboud University Nijmegen Medical Centre, P.O. Box 9101, 6500 HB Nijmegen, The Netherlands; 3 Einstein Center for Digital Future, Wilhelmstraße 67, 10117 Berlin, Germany

**Keywords:** Cancer imaging, Cancer prevention, Oral cancer

## Abstract

Oral squamous cell carcinoma (OSCC) is amongst the most common malignancies, with an estimated incidence of 377,000 and 177,000 deaths worldwide. The interval between the onset of symptoms and the start of adequate treatment is directly related to tumor stage and 5-year-survival rates of patients. Early detection is therefore crucial for efficient cancer therapy. This study aims to detect OSCC on clinical photographs (CP) automatically. 1406 CP(s) were manually annotated and labeled as a reference. A deep-learning approach based on Swin-Transformer was trained and validated on 1265 CP(s). Subsequently, the trained algorithm was applied to a test set consisting of 141 CP(s). The classification accuracy and the area-under-the-curve (AUC) were calculated. The proposed method achieved a classification accuracy of 0.986 and an AUC of 0.99 for classifying OSCC on clinical photographs. Deep learning-based assistance of clinicians may raise the rate of early detection of oral cancer and hence the survival rate and quality of life of patients.

## Introduction

Oral squamous cell carcinoma (OSCC) is among the most common malignancies worldwide, with a reported incidence of 377,713 and 117,757 deaths in 2020^1^. The five-year survival rate is over 80% in the early stages, decreasing to < 30% for advanced disease. More than 60% of the OSCCs are diagnosed at an advanced stage with high morbidity and mortality^2–4^. The incidence and mortality rates underline the importance of oral cancer screening programs to improve early detection and therapeutic success^5–7^.

Although the golden standard is pathologically proven, early detection can be achieved visually as OSCCs start superficially from squamous cell metaplasia. Nonetheless, the diagnostic accuracy of primary health care professionals is limited, with a sensitivity of 57.8% and a specificity of between 31 and 53%^8,9^. The lack of adequate training, substantial heterogeneity, and the lack of experience impede an effective diagnosis by primary health care professionals^6,10^. An automated assistance system may improve the diagnostic accuracy, allowing a more reliable and accurate assessment of the oral cavity, especially in the hands of less experienced professionals.

With advancements in artificial intelligence, deep learning algorithms have been adopted in computer-aided detection and diagnosis (CAD). Mainly convolutional neural networks (CNN) have emerged as the state-of-the-art approach to medical image analysis. CNN's utilize convolutional kernels with small perceptual fields to extract features via weight sharing and local connectivity^11^. Recently, transformers have been introduced as an alternative approach to CNNs. Transformers are based on an attention mechanism that efficiently estimates each pixel-pair interplay^12^.

In oral and maxillofacial surgery, few studies have explored the capability of CNNs to automatically classify OSCC on clinical photographs. These studies addressed the classification and detection of oral potentially malignant diseases^13^ and oral cancer lesions^5,14,15^ using YOLOv5, ResNet-152, DensNet-161, Inception-v4 and EfficientNet-b4, respectively.

However, none of the studies has explored the accuracy of vision transformers for classifying OSCC. This study aims to develop an automated oral cancer screening system using vision transformers as a fundamental basis for a timely and accurate referral system.

## Material and methods

### Data

In the present study, 1406 clinical photographs (CPs) were randomly collected from the Department of Oral and Maxillofacial Surgery, Charité - Universitätsmedizin Berlin, Germany (mean age of 60.8 years, age range of 15–90 years). The photographs were acquired with single lens reflex (SLR) cameras with varying light exposures. The image resolution was a minimum of 72 dpi. CPs with masked lesions or foreign bodies (e.g., mirrors or tongue depressors) were excluded from further analyses as described in a previous study^16^. All image data were anonymized and de-identified before analysis. Informed consent for the analysis of data was obtained from all patients or their guardians in case of age below 18 years. This study has been conducted in accordance with the code of ethics of the World Medical Association (Declaration of Helsinki). The approval of this study was granted by the Institutional Review Board, the Ethics Committee of Charité – Universitätsmedizin Berlin (EA2/089/22).

### Data classification

Different clinicians verified all CPs based on electronic medical records (EMR). CPs with OSCCs needed to be biopsy-proven. All CPs were subsequently reviewed and revised by three clinicians (RG, DT, TF). The three reviewers have at least five years of clinical experience. Each clinician was instructed and calibrated in the verification task using a standardized protocol before the selection and reviewing process. The final dataset consisted of 703 CPs of OSCC and 703 CPs of normal oral mucosae (Table 1).

**Table 1:** Baseline characteristics of the malignant pathologies.

Entity	Number of images	Percentage
OSCC	638	90.7
Verrucous SCC	23	3.3
Sarcomatoid SCC	4	0.6
Carcinoma in situ	13	1.9
OSCC (clinical)	25	3.5
Gender		
Male	436	62
Female	267	38
Location*		
Tongue	258	36.91
Floor of mouth	208	29.64
Maxilla	22	3.17
Mandible	111	15.75
Buccal mucosa	95	13.61
Palate	7	0.65
Oropharynx	2	0.28
Staging		
Tis	7	1.06
T1	203	28.9
T2	191	27.2
T3	115	16.3
T4	152	21.6
T unknown	35	5.0
Grading		
G1	102	14.5
G2	386	55
G3	143	20.3
G4	3	0.5
G unknown	69	9.7

Multiple locations of extended lesions possible (*).

The normal tissue dataset comprised photographs of the oral cavity without premalignant oral mucosal lesions or oral cancer. A further selection of the dataset to exclude possible anatomical variations or inflammatory conditions of the gingiva or mucosa was not performed.

The OSCC training dataset contained images of various tumor stages, including Tis (1%), T1 (28.9%), T2 (27.2%), T3 (16.3%), T4 (21.6%) and unknown tumor stages (5%). The locations were tongue (36.9%), floor of mouth (29.6%), maxilla (3.2%), mandible (15.8%), buccal mucosa (13.6%), palate (0.7%) and oropharynx (0.3%). The test and validation data sets contained a comparable distribution of tumor stages and locations with a maximum deviation of 10% from the training dataset.

### The model

**Figure 1:**
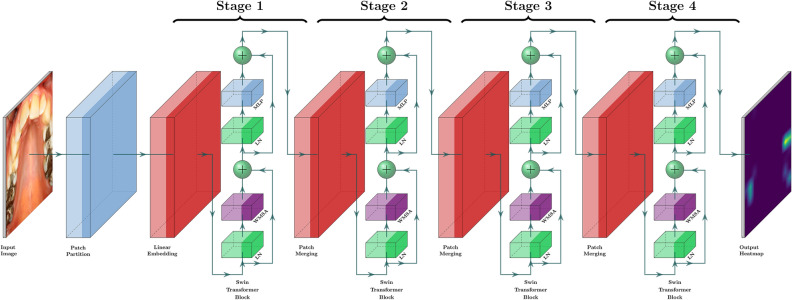
Swin-transformer network.

The Swin-Transformer was used in this study^17^. This transformer is characterized by its shift of the window partition between consecutive self-attention layers. The shifted windows connect with preceding layers’ window, increasing the modelling power efficiently. The employed model is shown in Fig. 1.

### Model training

The annotated were randomly divided into 3 sets of CPs, 1124 for training, 141 for validation and 141 for testing. The validation set was used to evaluate the model performance during training, while the hold-out test set was used to evaluate the model performance after training.

The Swin-Transformer was pre-trained on the ImageNet dataset and optimized using a stochastic gradient descent with a learning rate of 5 × 10^–3^, a momentum of 0.9 and a weight decay of 1 × 10^–4^. No gradient clipping was applied. The model was implemented in PyTorch 1.11.0 and trained on a 12 GB NVIDIA TITAN V GPU. Model training was previously described in a study on caries detection radiographs^16^.

### Statistical analysis

The transformer predictions on the test set were compared to the histopathological ground truth. Classification metrics are reported as follows for the test set: accuracy $= \frac{TP+TN}{TP+TN+FP+FN}$, positive predictive value $= \frac{TP}{TP+FP}$, F1-score $= \frac{2TP}{2TP+FP+FN}$ , sensitivity $= \frac{TP}{TP+FN}$ , specificity $= \frac{TN}{TN+FP}$ , negative predictive value $= \frac{TN}{TN+FN}$. TP, TN, FP, and FN denote true positives, true negatives, false positives, and false negatives, respectively. Furthermore, the area-under-the-curve-receiver-operating-characteristics-curve (AUC) and confusion matrix are presented. Gradient-weighted Class Activation Mapping (Grad-CAM), a class-discriminative localization technique was applied, to generate visual explanations highlighting the important regions on CPs for classifying OSCC. Statistical analysis was performed as in a previous study^16^.

## Results

**Figure 2:**
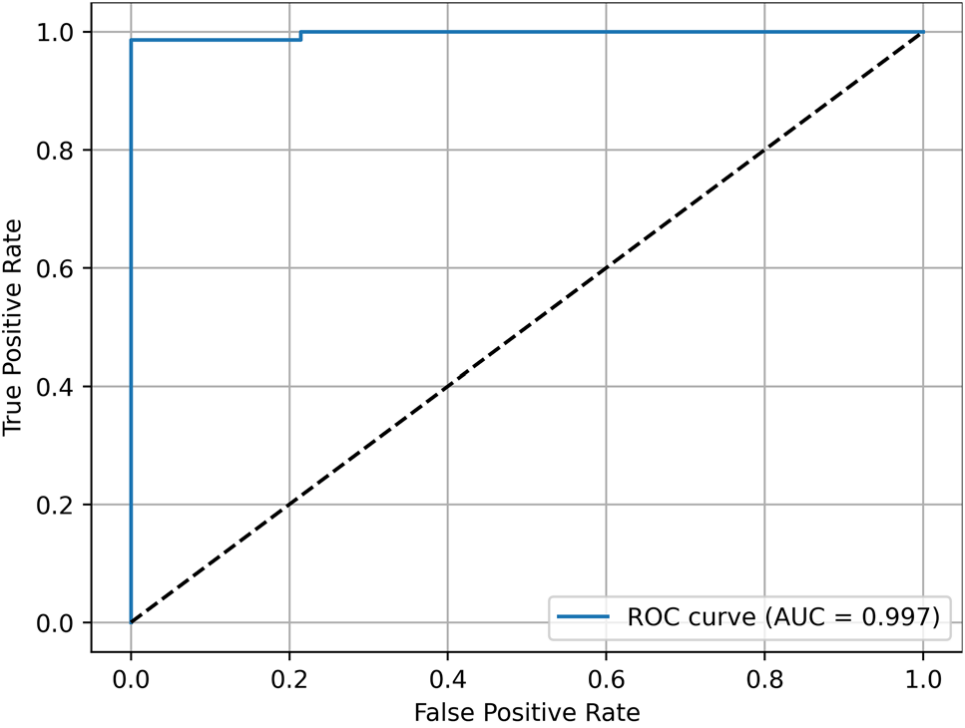
Area-under-the-curve-receiver-operating-characteristics-curve. The ROC is created by plotting the true positive against the false positive rate at different thresholds.

**Figure 3:**
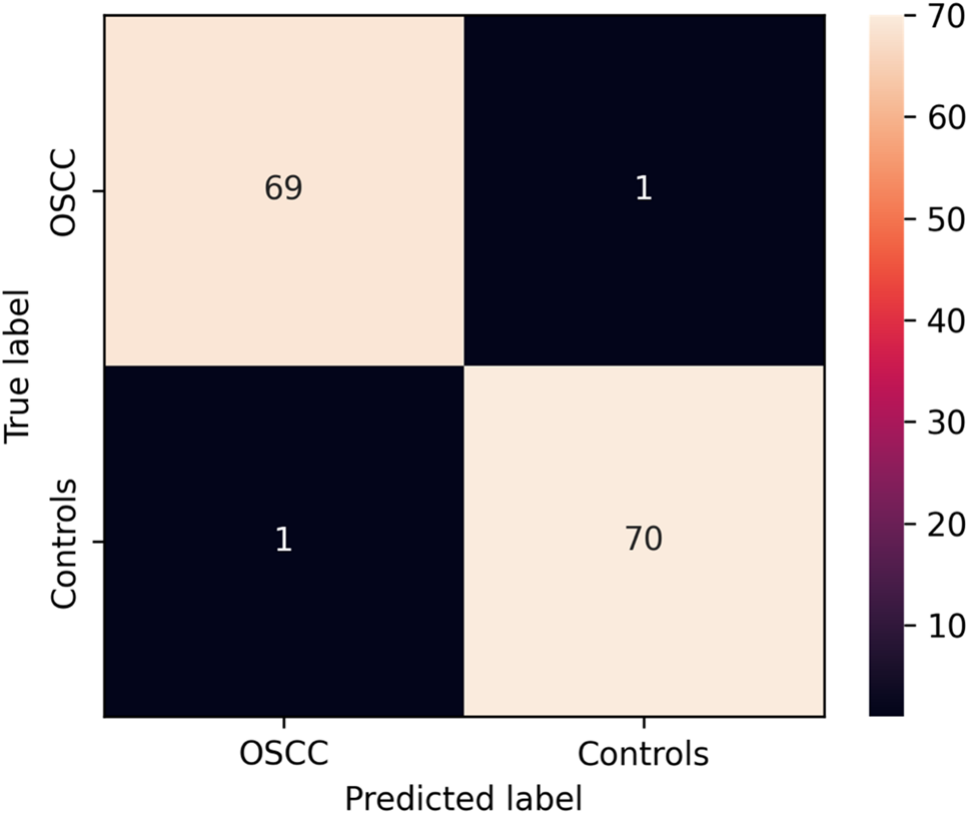
Confusion matrix illustrating the binary classification results.

Table 2 summarizes the classification performance of the Swin-Transformer on the test set, including the accuracy, positive predictive value, sensitivity, specificity and negative predictive value. The classification accuracy was 98,6%. The model achieved an AUC of 0.99 (Fig. 2). The confusion matrix is presented in Fig. 3.

**Table 2:** The Accuracy, positive predictive value (PPV), F1-score, sensitivity, specificity and negative predictive value (NPV) for the detection of OSCC on CP.

Accuracy	PPV	F1-score	Sensitivity	Specificity	NPV
0.9858	0.9857	0.9857	0.9857	0.9859	0.9859

**Figure 4:**
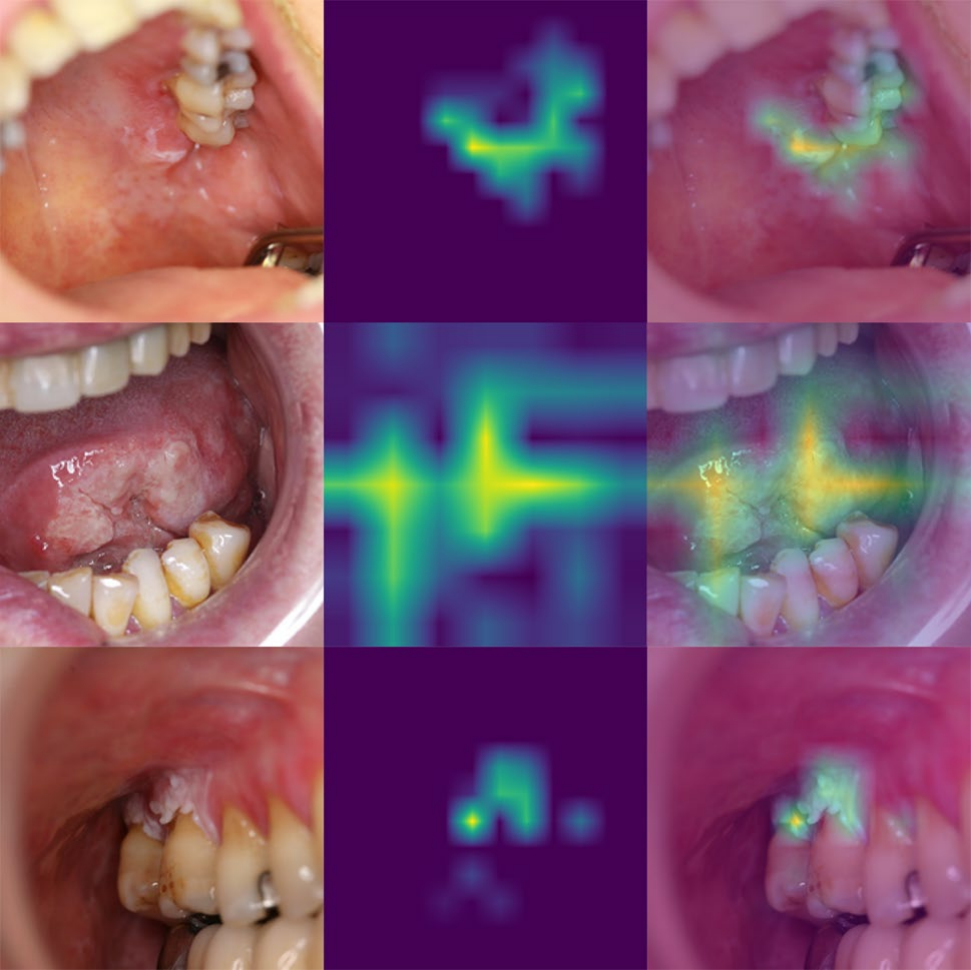
Class activation map for OSCC. The left column shows the CP of OSCC. The middle column represents the class activation map. The right column illustrates the overlay of CP and activation map.

**Figure 5:**
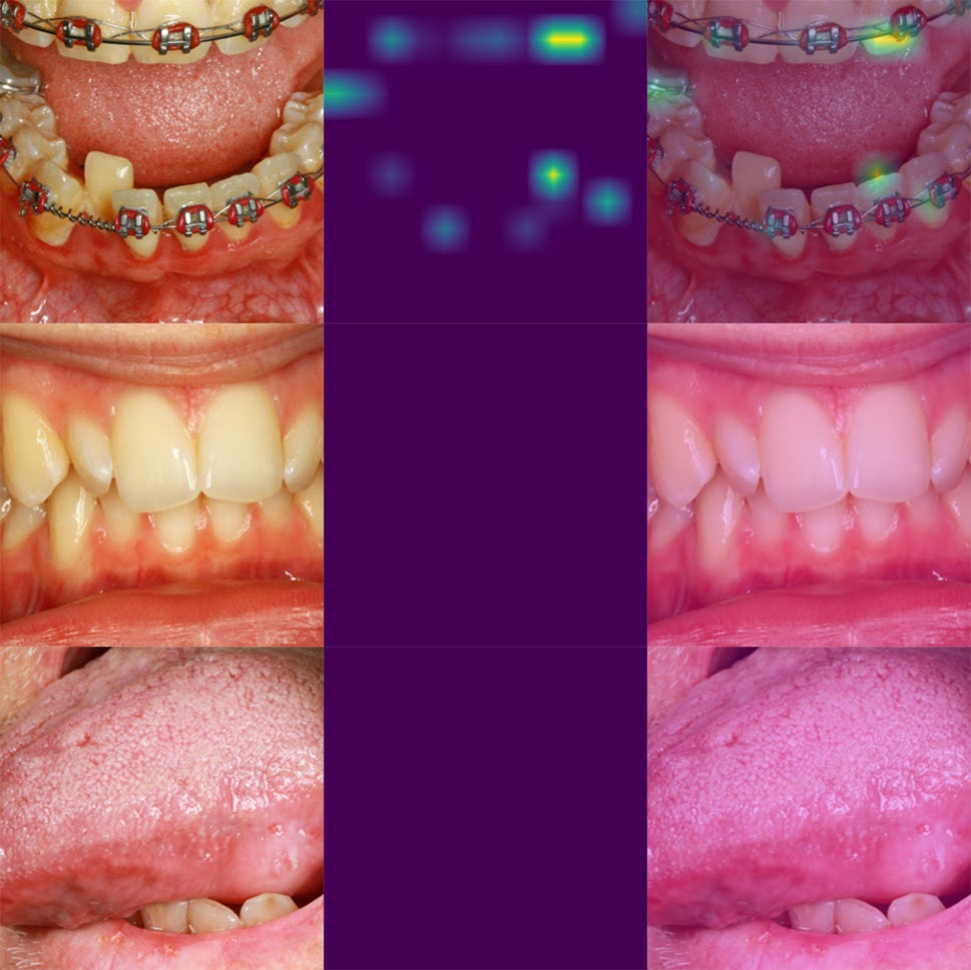
Class activation map for normal mucosa. The left column shows the CP of normal mucosa. The middle column represents the class activation map. The right column illustrates the overlay of CP and activation map.

The class activation heatmaps (CAM) of OSCC and normal oral mucosae are illustrated in Figs. 4 and 5. These heatmaps visualize the discriminative regions used by the Swin-Transformer for the classification. Optical inspection indicates a more centered and focused region of interest for OSCC. For normal mucosa, either a blank heatmap without any focus or a widely distributed focus was noticed.

## Discussion

Oral squamous cell carcinoma is a common malignancy with overall high mortality and morbidity^2–4^. The lack of experience and training of primary health care professionals leads to diagnostic delays and consequently to more extensive surgical procedures with more extended hospitalization and lower survival rates^18–20^. An automated assistance system for the clinician may increase the diagnostic accuracy while reducing the observer dependency. The vision transformer network introduced in this study is an accurate tool for the classification of OSCC based on clinical photographs that are most often acquired for documentation purposes.

In the past, diagnostic methods such as vital staining, autofluorescence and chemiluminescence, narrow band imaging, and optical spectroscopy have been introduced and documented with varying sensitivity and specificity^21^. Vital staining had a sensitivity of 92.3% but most studies did not report the specificity. For autofluorescence, heterogeneous values of 50–100% for sensitivity and 12.5 and 75.5% for specificity were reported. Narrow band imaging showed high sensitivity between 84.62 and 93.93%, and specificity between 75.7 and 94.56%.

Different studies have recently applied CNNs to classify oral cancer from an oral photograph. Warin et al. reported a F1-score of 0.9875 and an AUC of 0.99 with DenseNet121^13^. Fu et al. achieved a similar F1-score (0.935–0.995) with a two-step approach^15^. In the first step, a Single Shot MultiBox Detector was applied to detect the region of interest. Subsequently, DenseNet assessed the pre-selected region of interest in the presence of OSCC. Welikala et al. achieved a significantly lower F1-score of 0.8707 with ResNet-101^22^. Shamim et al. compared multiple CNNs (e.g. AlexNet, GoogleNet, Inceptionv3, ResNet50, SqueezeNet and VGG19) to classify tongue lesions and achieved F1-scores ranging from 0.9048 to 0.9756^23^.

However, a direct comparison of these previous studies should be regarded with caution. The performance of the CNNs is highly dependent on the dataset, the hyperparameters and the architecture itself^24^. The number of training and test sets varied greatly in the previous studies, and the data representativeness was unclear. Furthermore, clinical photographs were not standardized, and a high discrepancy was expected in perspective. For these reasons, the replication and validation of the previous results remain impracticable.

In the current study, the Swin-Transformer achieved a F1-score of 0.98 and an AUC of 0.99. The model had one false positive prediction and one false negative prediction, independent of location, staging or grading. Two key concepts are essential for high performance: hierarchical feature maps and shifted window attention. Firstly, hierarchical feature maps allow the intermediate tensors to be merged from layer to layer, reducing the spatial dimension (i.e. downsampling) of the feature maps effectively. In comparison to CNNs, patch merging is applied for downsampling instead of convolution operations. Secondly, the Swin-Transformer replaced the standard multi-head self-attention with a window and shifted window self-attention. The standard multi-head self-attention performs a global self-attention, resulting in a quadratic complexity. For this reason, the window self-attention computes attention only locally within specified windows. The shifted window self-attention addresses global information loss using cross-window connections between different layers. These two modules reduce the quadratic complexity to linear complexity^17^.

Although a high performance was achieved using a transformer, there are limitations. The reported study is limited by its monocentric design resulting in a database consisting of the local population. The photographic images were acquired with high-quality cameras and did not regard clinical settings in which images may be acquired with cameras or mobile devices with lower image quality. The Swin-Transformer are strictly confined to the employed train- and test set and may perform worse in real-world scenarios. Prospective studies are required to evaluate the diagnostic accuracy of the Swin-Transformer in a clinical setting.

In conclusion, the Swin-Transformer forms a promising foundation for further developing automatic screening of OSCC on clinical photographs. Deep learning-based assistance of clinicians may raise the rate of early detection of oral cancer and hence the survival rate and quality of life of patients.

## Data Availability

The datasets analyzed in the current study are not publicly available due to data protection but are available from the corresponding author on reasonable request.
